# Superbugs *Neisseria gonorrhoeae* und *Mycoplasma genitalium*: Therapeutische Herausforderungen durch antimikrobielle Resistenzen

**DOI:** 10.1007/s00105-026-05650-0

**Published:** 2026-03-02

**Authors:** Angelika Kogler, David Chromy, Birgit Sadoghi

**Affiliations:** 1 Department of Dermatology and Venereology, Medical University of Graz, Auenbruggerplatz 8, 8036 Graz, Österreich; 2 Department of Dermatology, Medical University of Vienna, Vienna, Österreich

**Keywords:** Sexuell übertragbare Infektionen, Multiresistente Stämme, Kombinierte Resistenzen, Reserveantibiotika, Resistenzentwicklung, Sexually transmitted infections, Multiple drug resistance, Combined antimicrobial resistance, Reserve antibiotics, Resistance development

## Abstract

Sexuell übertragbare Infektionen (STI) nehmen deutlich zu. Die Inzidenz von Infektionen mit *Neisseria gonorrhoeae* (NG) ist laut den rezentesten ECDC-Daten in den letzten 10 Jahren um mehr als 300 % angestiegen. Außerdem nehmen Resistenzen sowohl bei NG und einem anderen STI-Erreger, *Mycoplasma genitalium* (MG), stetig zu. Multiresistente Stämme („Superbugs“) stellen im klinischen Alltag eine größer werdende Herausforderung dar. Im Folgenden wird eine Literaturübersicht rezenter Studien und aktueller Leitlinien vorgestellt. NG zeigt weltweit hohe Resistenzraten gegenüber Penizillinen, Tetracyclinen und Fluorchinolonen sowie zunehmend auch eine reduzierte Empfindlichkeit gegenüber dem Makrolidantibiotikum Azithromycin. Die Resistenzraten gegenüber Ceftriaxon, der derzeitigen Erstlinientherapie, sind in Europa weiterhin sehr gering. Im südostasiatischen Raum wurde zuletzt jedoch ein deutlicher Anstieg von Ceftriaxon-Resistenzen bei NG beobachtet. Bei MG werden global steigende Resistenzen v. a. gegenüber Makroliden und seltener Fluorchinolonen beobachtet. Eine kombinierte Resistenz von MG gegen beide Wirkstoffklassen ist keine Seltenheit mehr und erfordert regelmäßig den Einsatz von Reserveantibiotika wie Pristinamycin oder Sitafloxacin. Zusammenfassend ist festzuhalten, dass die zunehmende Resistenzentwicklung bei NG und MG ein relevantes globales Gesundheitsproblem darstellt. Verstärkte Überwachung, molekulare Resistenzdiagnostik und gezielter Ansatz sowohl von Testungen als auch der Antibiotikatherapie sind entscheidend für eine nachhaltige Infektionskontrolle.

## Abstract (en)

The incidence of sexually transmitted diseases continues to rise. According to the most recent European Centre for Disease Prevention and Control (ECDC) data, infections with *Neisseria *(*N.*)* gonorrhoeae* have increased by more than 300% over the past 10 years. The growing antimicrobial resistance of *N. gonorrhoeae* and *Mycoplasma *(*M.*)* genitalium* is leading to infections that are increasingly difficult to treat. Multidrug-resistant strains (“superbugs”) cause persistent infections and increased transmission. Thus, a review of recent studies and current guidelines was conducted. *N. gonorrhoeae* is showing increasing resistance to penicillins, tetracyclines, and fluoroquinolones; furthermore, there is a growing reduction in susceptibility to the macrolide antibiotic azithromycin. Resistance rates to ceftriaxone, the current first-line therapy in many international treatment guidelines, and to cefixime, the peroral treatment option frequently used in outpatient settings, remain very low in German-speaking countries. For *M. genitalium*, increasing resistance is observed primarily to macrolides and, less commonly, to fluocinolones; cases of infections with combined resistance to both antimicrobial classes have been reported and regularly require the use of reserve antibiotics such as pristinamycin or sitafloxacin. Enhanced surveillance, molecular resistance testing, avoidance of overdiagnosis, rational and responsible antibiotic use (preventing overtreatment), and the development and implementation of new antimicrobial agents are crucial for sustainable infection control.

Die Inzidenz bakterieller sexuell übertragbarer Infektionen (STI) ist in den letzten Jahren deutlich angestiegen [1, 2]. Vor allem bakterielle STIs nehmen klar zu: So zeigten die rezentesten ECDC-Daten einen Anstieg der Syphilisinzidenz um 100 % in den letzten 10 Jahren und einen Anstieg der Gonorrhöinzidenz um 321 % [3, 4]. Nicht nur Männer, die Sex mit Männern haben (MSM), sind überproportional betroffen, sondern auch bei Männern, die Sex mit Frauen haben, und Frauen wurde ein Anstieg beobachtet. Die Zahl der Syphilisfälle bei Frauen ist in Europa in den letzten Jahren deutlich gestiegen [5]. Bei der Gonorrhö gab es bei Frauen eine Steigerung um mehr als 200 %, wobei die Gruppe der unter 25-Jährigen am stärksten betroffen war. Zunehmende Resistenzen von *Neisseria gonorrhoeae* (NG) und *Mycoplasma genitalium* (MG) gegenüber den bisher verwendeten Standardantibiotika führen immer häufiger zu Therapieversagen und in weiterer Folge zu verlängerter Infektiosität und erhöhter Transmission [6–8]. Damit einher geht das vermehrte Auftreten sog. Superbugs: Bakterien, die gegen mehrere Antibiotikaklassen gleichzeitig resistent sind und daher mit den üblichen antimikrobiellen Therapien nur schwer oder gar nicht behandelbar sind.

## Überblick *Neisseria gonorrhoeae *und *Mycoplasma genitalium*

### *Neisseria gonorrhoeae*

*Neisseria gonorrhoeae, *ein gramnegativer Diplokokkus, ist mit einer geschätzten Inzidenz von 86,9 Mio. Neuinfektion pro Jahr weltweit nach *Chlamydia trachomatis *der zweithäufigste sexuell übertragbare bakterielle Erreger [9]. Die Inkubationszeit gilt als sehr kurz; typischerweise 1 bis 3 Tage. Die Übertragung erfolgt nahezu ausschließlich durch Sexualkontakte. Bei der Gonoblenorrhö sind heutzutage jedoch Autoinokulationen die häufigste Infektionsquelle.

**Abb. 1:**
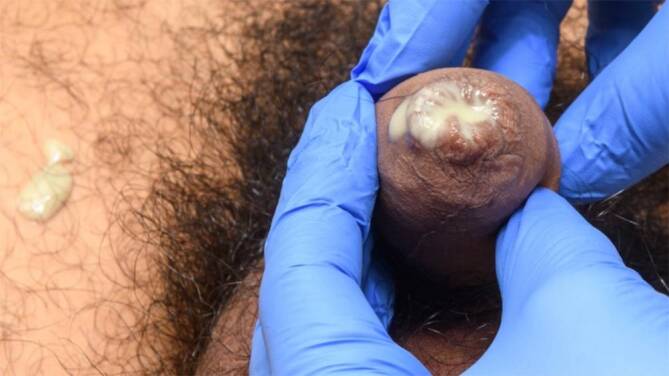
Purulenter urethraler Ausfluss beim Mann bei Gonokokkeninfektion. (Mit freundl. Genehmigung, © B. Sadoghi, Medizinische Universität Graz)

Die typische Klinik ist der purulente urethrale Fluor beim Mann (Abb. 1) und die purulente Zervizitis der Frau. Bei Nichtbehandlung des Mannes kann es zu aufsteigenden Infektionen im Sinne einer Urethritis posterior, Epididymitis oder Epididymoorchitis kommen. Bei Nichtbehandlung der Frau kann eine Salpingitis, Endometritis oder gar Perihepatitis gonorrhoica resultieren. Das ausschließlich bei Frauen vorkommende Bild einer „pelvic inflammatory disease“ (PID) zeichnet sich durch akut einsetzende starke Unterbauchschmerzen aus und wird in über 85 % der Fälle durch STI-Erreger, v. a. *Chlamydia trachomatis*, NG, aber auch MG verursacht [10]. Da eine PID in einer wesentlichen Morbidität resultieren kann (ektope Schwangerschaft, chronische Schmerzsymptomatik, tubare Infertilität durch Reduktion der tubaren Zilien), ist dringend eine rasche Therapie (initial Triple-Antibiose) mit Ceftriaxon, Doxycyclin und Metronidazol angeraten [10].

​

Extragenitale Infektionen im Sinne einer NG-Proktitis oder NG-Pharyngitis sind möglich und bleiben bei beiden Geschlechtern häufig unbemerkt [11]. Urogenitale Infektionen verlaufen bei Frauen in 86–93 % asymptomatisch; bei Männern hingegen verlaufen urethrale Infektionen in 90 % der Fälle symptomatisch [12, 13]. Unbehandelte Infektionen können bei hämatogener Streuung – insbesondere in gewissen klinischen Situationen wie einer bestehenden Immunschwäche – zu einer disseminierten Gonokokkeninfektion führen [14]. Zudem erhöht eine Infektion mit NG das Risiko einer Akquisition einer HIV-Infektion [15].

Typische Klinik ist der purulente urethrale Fluor beim Mann und die purulente Zervizitis der Frau

**Abb. 2:**
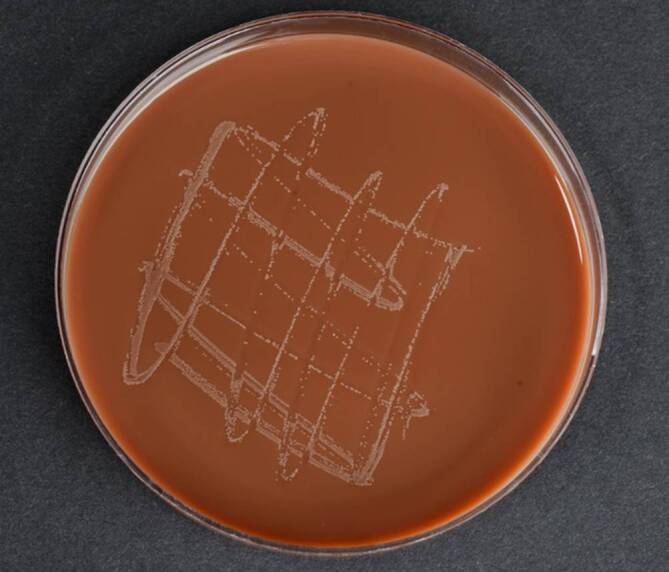
Kultur von *Neisseria gonorrhoeae *zur Durchführung einer Resistenztestung. (Mit freundl. Genehmigung, © B. Sadoghi, Medizinische Universität Graz)

Der Nachweis von NG erfolgt aufgrund der hohen Sensitivität und Spezifität für urogenitale und extragenitale Proben in erster Linie mittels Nukleinsäureamplifikationstests (NAATs). Der Nachweis von intrazellulären Diplokokken in Leukozyten in Gram- und Methylenblau-gefärbten Ausstrichpräparaten ist bei symptomatischen Männern mit Urethritis sehr spezifisch und sensitiv; bei extragenitalen Infektionen sowie bei asymptomatischen Personen hat die Ausstrichdiagnostik keinen Stellenwert [12, 16]. Zur antimikrobiellen Resistenztestung wird die Durchführung einer Kultur bei NG (Abb. 2) dringend empfohlen [17]. Bei MG ist aus biologischen Gründen eine Kultur zur phänotypischen Resistenzanalyse nicht möglich.

#### Wann ist ein Screening empfohlen?

Die CDC empfiehlt eine Testung auf NG bei symptomatischen Patient*innen sowie ein jährliches Screening auf NG für Frauen unter 25 Jahre, MSM und für Personen mit häufig wechselnden Sexualpartner*innen [16]. Menschen mit HIV sollten generell 1‑mal jährlich auf STIs untersucht werden. Für Schwangere mit erhöhtem Risiko wird ein Screening beim ersten pränatalen Besuch und ggf. im dritten Trimenon empfohlen [16]. Bei MSM mit sehr hohem Risiko wird laut aktuellen Leitlinien ein Screening alle 3 bis 6 Monate empfohlen [16]. Die optimale Screeningfrequenz für NG bei MSM, die unter Präexpositionsprophylaxe (PrEP) stehen, wird derzeit kontrovers diskutiert [18]. Ursprünglich wurde empfohlen, alle 3 bis 6 Monate an 3 anatomischen Lokalisationen zu screenen. Die Gonoscreen-Studie, die MSM und Transfrauen unter PrEP einschloss, zeigte, dass ein vierteljährliches Screening an 3 anatomischen Lokalisationen (urethral, rektal, pharyngeal) zwar die Inzidenz von *Chlamydia trachomatis* (CT) senkt, jedoch keinen signifikanten Einfluss auf die Inzidenz von NG hat [19]. Häufiges Screening war mit einem erhöhten Antibiotikaverbrauch und somit einem potenziell gesteigerten Risiko für Resistenzentwicklung verbunden, ohne dass schwerwiegende Komplikationen oder relevante Unterschiede im Auftreten symptomatischer NG-Infektionen beobachtet wurden [19]. Als Konsequenz wurde das routinemäßige vierteljährliche Screening in Belgien aus den nationalen Leitlinien gestrichen [20]. Um hier „overdiagnosis“ und konsekutives „overtreatment“ zu vermeiden, wird die optimale Screeningfrequenz derzeit stark diskutiert.

### *Mycoplasma genitalium*

*Mycoplasma genitalium* ist ein atypisches, sehr kleines, parasitäres Bakterium ohne Zellwand. Dies erschwert einerseits die Diagnostik und schränkt andererseits das Spektrum möglicher Antibiotika ein. Die Inkubationszeit ist deutlich länger als bei Gonorrhö und wird laut Literatur nicht einheitlich mit ca. 4 bis 8 Wochen angegeben. Die Prävalenz in der Allgemeinbevölkerung gilt als gering und liegt bei etwa 1–3 %, ist jedoch in bestimmten Gruppen wie bei MSM mit 10,5 % deutlich höher [21–23].

*Mycoplasma genitalium *ist bei Männern für 30–40 % der persistierenden oder rezidivierenden nichtgonorrhoischen Urethritiden verantwortlich [12, 24]. Kommt es zu einem sichtbaren Fluor, ist dieser meist serös-gelblich, jedoch nicht rahmig oder purulent. Bei Frauen ist MG mit Zervizitis, PID, Infertilität sowie möglicherweise mit Schwangerschaftskomplikationen assoziiert [12, 16].

Rektale MG-Infektionen treten insbesondere bei MSM häufig auf und verlaufen meist asymptomatisch [25]. Der Zusammenhang zwischen rektalen MG-Infektionen und dem Auftreten klinischer Symptome im Sinne proktitischer Beschwerden ist bislang nicht eindeutig geklärt; eine rektale Transmission ist jedoch möglich [25]. In einer aktuellen multizentrischen Studie aus Belgien, Deutschland, Spanien und Großbritannien lag die Prävalenz rektaler MG-Infektionen mit 12,5 % deutlich über jener urethraler Infektionen (3,9 %) [26].

Die klinische Relevanz von MG als urogenitales Pathogen ist mittlerweile weitgehend anerkannt. Aufgrund der hohen Prävalenz asymptomatischer und teils selbstlimitierender Verläufe sowie der schwierigen Therapie infolge weitverbreiteter antimikrobieller Resistenzen besteht jedoch Uneinigkeit hinsichtlich des klinischen Managements. Diskutiert werden insbesondere das Screening asymptomatischer Risikogruppen, die routinemäßige molekulardiagnostische Testung bei symptomatischer Urethritis sowie die Behandlungsbedürftigkeit asymptomatischer MG-Infektionen [17]. Eine australische Arbeit konnte in einer Modellierung darlegen, dass bei einer MG-Ausgangsprävalenz von 9,4 % bei MSM, die nur getestet werden, sofern Beschwerden bestehen, eine Reduktion der Prävalenz auf 6,4 % möglich wäre, wenn ein MG-Screening bei allen MSM durchgeführt würde. Kalkuliert wurde damit, dass alle diagnostizierten Infektionen eine Therapie erhalten. Ebenfalls konnten die Autoren dieser Arbeit zeigen, dass diese Reduktion der Hintergrundprävalenz zu einem signifikanten Anstieg von Makrolidresistenzen führen würde [27].

Zum Nachweis von MG sind ausschließlich NAATs geeignet. Bei positivem Nachweis ist eine molekulare Resistenztestung zur Therapieentscheidung indiziert, sofern verfügbar [28, 29].

#### Wann ist ein Screening empfohlen?

Im Gegensatz zu NG wird ein routinemäßiges Screening von MG bei asymptomatischen Risikogruppen nicht empfohlen, da die klinische Relevanz asymptomatischer Infektionen unklar ist und ein breites Screening zu unnötiger Antibiotikatherapie und folglich Resistenzentwicklung führen könnte. Eine Testung auf MG sollte laut Leitlinien ausschließlich bei symptomatischen Personen (insbesondere bei Männern mit persistierender oder rezidivierender nichtgonorrhoischer Urethritis [NGU] sowie bei Frauen mit persistierender Zervizitis oder bei Verdacht auf PID) und deren Sexualpartnern erfolgen [6, 12, 16, 30]. Auch eine rektale Testung ist ausschließlich bei symptomatischer Proktitis nach Ausschluss von NG und CT indiziert [25].

## Resistenzentwicklung

Als „drug-resistant“ bzw. als „antibiotikaresistent“ wird die erworbene Unempfindlichkeit eines bakteriellen Isolats gegenüber mindestens einem antimikrobiellen Wirkstoff bezeichnet, der zur Behandlung der jeweiligen Infektion eingesetzt wird [31]. Je nach Ausprägung der Resistenz wird zwischen 3 Begriffen unterschieden:

- „multidrug-resistant“ (MDR) bedeutet, dass ein bakterielles Isolat gegen mindestens einen Wirkstoff in 3 oder mehr Antibiotikaklassen nicht empfindlich ist;
- von „extensively drug-resistant“ (XDR) spricht man, wenn ein Isolat nur noch gegen 1 oder 2 Wirkstoffklassen empfindlich ist, und
- „pandrug-resistant“ (PDR) beschreibt Isolate, die gegen alle Wirkstoffklassen unempfindlich sind [31].

### *Neisseria gonorrhoeae*

In der aktuellen World Health Organization (WHO) Bacterial Priority Pathogen List 2024 wird NG aufgrund zunehmender Resistenz als „high priority“ eingestuft [32]. Dies unterstreicht die zunehmende globale Bedeutung antimikrobieller Resistenzen (AMR) bei NG und den dringenden Bedarf neuer Therapieoptionen.

*Neisseria gonorrhoeae* zeigt weltweit – und so auch im deutschsprachigen Raum – eine hohe Resistenz gegenüber früher zur Behandlung der Gonorrhö eingesetzten Antibiotika wie Ciprofloxacin, Tetracyclin und Penizillin. Für Deutschland wurde im Jahr 2022 eine Ciprofloxacin-Resistenz von 65,8 % und eine Penizillin-Resistenz von etwa 30–80 % berichtet, wobei Tetracyclin-Resistenzen in aktuellen europäischen Surveillance-Daten ebenfalls sehr hoch sind (bis zu 96 % in einzelnen Kohorten) [33, 34]. Besonders besorgniserregend ist die Entwicklung der Resistenzraten gegen das Makrolidantibiotikum Azithromycin: Während in Deutschland im Jahr 2014 1,3 % der Isolate eine verminderte Empfindlichkeit (definiert durch eine minimale Hemmkonzentration > 1 mg/l) gegen Azithromycin aufwiesen, stieg dieser Prozentsatz laut RKI-Surveillance-Daten (Go-Surv-AMR) im Jahr 2023 auf einen Höchststand von 26,2 % mit einem Rückgang – laut vorläufigen Analysen – auf 16,7 % im Jahr 2024 [35]. Ähnliche Trends zeigen sich auch in Österreich, wo die Azithromycin-Resistenz von 2 % im Jahr 2013 auf 12 % im Jahr 2020 zugenommen hat [36]. Im Gegensatz dazu bleiben die Resistenzraten gegenüber Ceftriaxon im deutschsprachigen Raum seit Jahren stabil auf einem niedrigen Niveau. Surveillance-Daten aus Deutschland zeigen, dass der Anteil an Ceftriaxon-resistenten Isolaten < 1 % liegt [36]. Auch Cefexim-Resistenzen treten nur sporadisch (zwischen 1 und 2 % der Isolate) auf [36]. An eine mögliche Cephalosporin-Resistenz sollte insbesondere bei typischer Klinik und ungeschützten Sexualkontakten im südostasiatischen Raum gedacht werden, da hier bereits Resistenzen von bis zu 30 % beschrieben wurden [37–40].

### *Mycoplasma genitalium*

Allem voran ist festzuhalten, dass die Kultivierung von MG quasi nicht möglich ist aufgrund der Biologie dieses Bakteriums. Die Anzucht des Bakteriums zu etablieren ist ein hochaufwendiger Prozess, der sich über Jahre ziehen kann und daher einzelnen Forschungseinrichtungen vorbehalten ist. Dadurch ist bei MG keine klassische phänotypische Resistenzbestimmung mittels minimaler Hemmkonzentration möglich. Tatsächlich definiert sich der Phänotyp bei MG zumeist durch das klinische Ansprechen. Das hat auch Konsequenzen für die genotypische Resistenzbestimmung, die nun mehr und mehr in der Klinik Verwendung findet: Hier wurden Mutationen beschrieben und definiert, die vorwiegend bei Personen mit Therapieversagen nach Makrolid oder Fluorchinolon gefunden wurden. Vereinfacht gesagt, geht man daher davon aus, dass jene Mutationen eine Resistenz bedingen [41]. Makrolidresistenzen sind insbesondere mit Punktmutationen im V‑Domänen-Bereich der 23S-rRNA assoziiert, v. a. mit den Substitutionen A2058G, A2058T und A2059G (nach *Escherichia-coli*-Nummerierung), die zu einem klinisch relevanten Wirkungsverlust von Azithromycin führen [42]. Fluorchinolon-Resistenzen betreffen primär die Chinolon-Resistenz-bestimmenden Regionen (QRDR) der Gene *parC* und *gyrA*. Klinisch relevant sind dabei v. a. Aminosäureaustausche im *parC*-Gen insbesondere S83I, S83R und D87N, die mit einem deutlich schlechteren Ansprechen auf Moxifloxacin assoziiert sind [42].

Die Rate der genotypischen Makrolidresistenz (Azithromycin) bei MG liegt in Deutschland aktuell bei 69 % der untersuchten Isolate, wobei die Makrolidresistenzrate bei MSM signifikant höher als bei MSW und Frauen ist [43]. In Österreich wurde eine Azithromycin-Resistenz von 68 % festgestellt, insbesondere bei Männern, Männern, die Sex mit Männern haben (MSM), und Menschen mit HIV [23].

Die Fluorchinolon-Resistenz (Moxifloxacin) beträgt in Deutschland etwa 25 % [43]. Eine europäische Metaanalyse bestätigt einen Anstieg der Fluorchinolon-Resistenz in der Region auf etwa 13 % im Zeitraum 2018 bis 2021 [44]. Duale Resistenzen (Makrolide und Fluorchinolone) werden in Europa bei 6,5 % der Isolate beobachtet [44].

## Therapie

### *Neisseria gonorrhoeae*

Alle großen Fachgesellschaften empfehlen Ceftriaxon als Therapie der ersten Wahl, wobei sie sich lediglich in den Dosierungen geringfügig unterscheiden (Tab. 1). Die aktuellen ÖGSTD-Leitlinien empfehlen eine Einmalgabe von Ceftriaxon 1 g intramuskulär oder intravenös [45]. Die Deutsche STI-Gesellschaft (DSTIG) empfiehlt die Einmalgabe von Ceftriaxon 1–2 g intramuskulär oder intravenös [46]. Eine empirische Kombinationstherapie mit Azithromycin wird aufgrund steigender Resistenzraten nicht mehr empfohlen [47–49]. Zudem sollte für eine Koinfektion mit Chlamydien präferenziell Doxycyclin eingesetzt werden. Als Option (v. a. bei tatsächlicher Penizillin-Allergie) wäre die Möglichkeit einer Monotherapie mit Azithromycin 2 g zu nennen – jedoch ausschließlich bei nichtpharyngealer Gonorrhö und unter Berücksichtigung der zunehmenden Resistenzsituation. Auch wenn Ceftriaxon-resistente NG-Isolate zunehmend beschrieben werden, liegen die minimalen Hemmkonzentrationen (MIC) in Europa in den meisten Fällen nur knapp oberhalb des Resistenz-Breakpoints (z. B. 0,125–0,25 mg/l). Nach Gabe von 1 g Ceftriaxon i.m. werden sehr hohe Serumspiegel erreicht, die bei urethralen Infektionen häufig ausreichen, um solche niedriggradig resistenten Stämme zu eradizieren. Deutlich problematischer sind hingegen pharyngeale NG-Infektionen, insbesondere jene, die mit 500 mg Ceftriaxon i.m. behandelt wurden [50, 51]. Die Tab. 1 zeigt einen Vergleich der Therapieempfehlungen verschiedener Fachgesellschaften (CDC, IUSTI, BASHH, ÖGSTD, DSTIG) für unterschiedliche Manifestationen der Gonorrhö (Tab. 1).

**Tab. 1:** Übersicht internationaler Leitlinienempfehlungen zur Therapie der Gonorrhö

Gesellschaft	Therapie Zervix/Urethra/Rektum	Alternative Therapie	Therapie Pharynx	Therapie Auge	Therapie disseminierte Infektion
*CDC*	Ceftriaxon 500 mg i.m. Ab 150 kg: 1000 mg	1) Gentamycin 240 mg i.m. + Azithromycin 2000 mg p.o. 2) Cefixim 800 mg p.o.	Ceftriaxon 500 mg i.m.	Ceftriaxon 500 mg i.m. + Spülung mit Kochsalzlösung	Ceftriaxon 1000–2000 mg i.v. alle 24 h für mindestens 7 Tage
*IUSTI*	Ceftriaxon 1000 mg i.m. + Azithromycin 2000 mg p.o.	1) Ciprofloxacin 500 mg 2) Gentamycin 240 mg i.m. + Azithromycin 2000 mg p.o.	Ceftriaxon 1000 mg i.m. + Azithromycin 2000 mg p.o.	Ceftriaxon 1000 mg i.m. + Azithromycin 2000 mg p.o. + regelmäßige Spülung mit Kochsalzlösung	Ceftriaxon 1000 mg i.v. alle 8–24 h für mindestens 7 Tage Alternativ: Cefotaxim 1000 mg i.v. alle 8 h oder Spectinomycin 2000 mg i.m. alle 12 h
*BASHH*	Ceftriaxon 1000 mg i.m.	1) Cefixim 800 mg i.m. + Azithromycin 2000 mg p.o. 2) Gentamycin 240 mg i.m. + Azithromycin 2000 mg p.o.	Ceftriaxon 1000 mg i.m.	Ceftriaxon 1000 mg i.m. + Cefuroxim 5 % Augentropfen stündlich	Ceftriaxon 1000 mg i.m. oder i.v. alle 24 h für mindestens 7 Tage
ÖGSTD*	Ceftriaxon 1000 mg i.m.	1) Cefixim 800 mg oral ED 2) Gentamycin 240 mg i.m. ED plus Azithromycin 2 g oral ED 3) Cefixim 400 mg oral ED plus Azithromycin 2 g oral ED 4) Azithromycin 2 g oral ED	Ceftriaxon 1000 mg i.m. oder i.v.	Ceftriaxon 1000 mg i.m. oder i.v. für 3 Tage	Ceftriaxon 1000–2000 mg i.m oder i.v./1-mal pro Tag/7 Tage bzw. 14 Tage bei Meningitis und 28 Tage bei Endokarditis
*DSTIG*	Ceftriaxon 1000–2000 mg i.v. oder i.m. + Azithromycin 1500 mg p.o.	1) Azithromycin 2000 mg p.o. 2) Cefixim 800 mg p.o. + Azithromycin 1500 mg p.o.	Ceftriaxon 1000–2000 mg i.v. oder i.m. + Azithromycin 1500 mg p.o.	Ceftriaxon 2000 mg i.v. oder i.m. über 3 Tage + Azithromycin 1500 mg p.o. + Lavage mit 0,9 %-Kochsalzlösung	Ceftriaxon 2000 mg i.v. alle 24 h ≥ 7 Tage (10 bis 14 Tage bei Meningitis und 4 Wochen bei Endokarditis) + einmalig Azithromycin 1500 mg p.o.

* Derzeit in Überarbeitung

Ein „Test of Cure“ (TOC) wird in jedem Fall empfohlen, um persistierende Infektionen und das Auftreten von antimikrobiellen Resistenzen zu erkennen [14]. Ein TOC kann mittels Kultur frühestens nach 72 h oder mittels PCR nach ca. 3 Wochen erfolgen. Bei einem positiven TOC ist in Europa eine kurzfristige Reinfektion bis heute die wahrscheinlichste Erklärung und eine nochmalige Therapie mit Ceftriaxon eine legitime Strategie.

Aufgrund der zunehmenden Resistenzentwicklung werden derzeit mehrere neue Antibiotika klinisch geprüft, von denen sich einige bereits in einem fortgeschrittenen Zulassungsprozess befinden.

Zoliflodacin, ein orales Spiropyrimidinetrione-Antibiotikum, das als Einzeldosis verabreicht wird, zeigte in Phase-2- und Phase-3-Studien eine hohe Wirksamkeit bei der Behandlung unkomplizierter urogenitaler und rektaler NG-Infektionen (mikrobiologische Heilungsrate > 96 %). Die Wirksamkeit bei pharyngealen Infektionen fiel geringer aus, erreichte zwar gewisse Eradikationsraten, war jedoch Ceftriaxon unterlegen [52]. Die Nebenwirkungsraten (meistens gastrointestinale Nebenwirkungen) waren gering.

Bei Gepotidacin handelt es sich um einen neuartigen oralen Triazaacenaphthylen-Topoisomerase-II-Inhibitor, ein „First-in-class-Antibiotikum“, das in der Phase-3-Studie EAGLE‑1 eine Nichtunterlegenheit gegenüber Ceftriaxon plus Azithromycin zeigte (Eradikationsrate > 90 % bei unkomplizierter Gonorrhö) [53]. Gepotidacin ist in den USA seit März 2025 von der FDA für unkomplizierte Harnwegsinfektionen zugelassen; eine Zulassung für NG-Infektionen wird erwartet [53]. Interessanterweise zeigt Gepotidacin in vitro auch eine Wirksamkeit gegenüber MG [54, 55].

Beide Wirkstoffe wurden Mitte Dezember von der FDA zur Behandlung der unkomplizierten urogenitalen Gonorrhoe zugelassen.

### *Mycoplasma genitalium*

Aufgrund seiner fehlenden bakteriellen Zellwand weist MG eine natürliche Resistenz gegenüber Betalaktamantibiotika auf. Für die Behandlung stehen im Wesentlichen 4 Antibiotikaklassen zur Verfügung: Makrolide (z. B. Azithromycin) als Erstlinientherapeutika, sofern eine Sensibilität besteht, Fluorchinolone (v. a. Moxifloxacin) als Zweitlinientherapie sowie Streptogramine (Pristinamycin, das aktuell ausschließlich in Frankreich erhältlich ist) und Tetracycline (Minocyclin; Doxycyclin in kombinierten Schemata) als Drittlinienoptionen. Sitafloxacin, ein Fluorchinolon, wurde bereits in Leitlinien wie der S3-Leitlinie für Urethritis als Off-label-Therapieoption nach vorherigen Therapien erwähnt [17].

In den meisten Leitlinien wird bei nichtgonorrhoischer Urethritis (NGU) die Gabe von Doxycyclin 100 mg 1‑0‑1 empfohlen. Eine Ausheilung bei alleiniger Therapie mit Doxycyclin ist zwar unwahrscheinlich, jedoch führt das Tetracyclin zu einer Reduktion der bakteriellen Last und begünstigt damit eine unmittelbare Folgetherapie mit Azithromycin oder Moxifloxacin. Diese sequenzielle Therapie wurde erstmalig von Kollegen aus Australien beschrieben und führt zu signifikant besseren Ansprechraten [56]. Dieses Schema wurde schließlich in die CDC-Leitlinien übernommen: Doxycyclin 100 mg oral 2‑mal täglich für 7 Tage, gefolgt von Moxifloxacin 400 mg oral 1‑mal täglich für 7 Tage [12]. Azithromycin soll alternativ zu Moxifloxacin eingesetzt werden, wenn eine Makrolidsensitivität durch molekulare Makrolidresistenztestung nachgewiesen ist [12]; diese ist im klinischen Alltag routinemäßig etabliert, jedoch nicht flächendeckend verfügbar. Der Stellenwert von genotypischen Fluorchinolon-Resistenztests ist bislang noch nicht eindeutig geklärt und daher im klinischen Routinebetrieb nicht etabliert [57]. Die S2k-Leitlinie zur Urethritis empfiehlt bei nachgewiesener Makrolidsensitivität Azithromycin in einer Gesamtdosis von 2,5 g (1000 mg am ersten Tag, gefolgt von 500 mg an den Tagen 2 bis 4). Diese Dosis ist in der aktualisierten Leitlinie deutlich höher als in älteren Leitlinien (Azithromycin 500 mg an Tag 1, gefolgt von 250 mg an den Tagen 2 bis 5) [17]. Aufgrund der hohen Makrolidresistenzraten und zunehmender Fluorchinolon-Resistenzen wird, sofern verfügbar, eine resistenzgesteuerte Therapie ausdrücklich empfohlen [16].

Die Tab. 2 gibt einen Überblick über internationale Leitlinienempfehlungen in Abhängigkeit von Makrolidsensibilität bzw. Makrolidresistenz.

**Tab. 2:** Übersicht internationaler Leitlinienempfehlungen zur Erstlinientherapie von urethraler MG-Infektionen in Abhängigkeit von Makrolidsensibilität bzw. Makrolidresistenz

Gesellschaft	Therapie der 1. Wahl (Makrolidresistenz oder keine Testung möglich)	Therapie der 1. Wahl (Makrolid-sensibel)
*CDC*	Doxycyclin 100 mg p.o. 2‑mal täglich für 7 Tage, danach Moxifloxacin 400 mg p.o. 1‑mal täglich für 7 Tage	Doxycyclin 100 mg p.o. 2‑mal täglich für 7 Tage, danach Azithromycin 1 g p.o. 1‑malig, gefolgt von 500 mg p.o. 1‑mal täglich für 3 Tage (insgesamt 2,5 g)
*IUSTI*	Doxycyclin 100 mg p.o. 2‑mal täglich für 7 Tage, danach Moxifloxacin 400 mg p.o. 1‑mal täglich für 7 Tage	1) Azithromycin 500 mg p.o. an Tag 1, danach 250 mg an Tag 2 bis 5 (Empfehlungsgrad 1B) 2) Josamycin 500 mg p.o. 3‑mal täglich für 10 Tage (Empfehlungsgrad 2C)
*BASHH*	Doxycyclin 100 mg 2‑mal täglich für 7 Tage, danach Moxifloxacin 400 mg 1‑mal täglich für 7 Tage	Doxycyclin 100 mg 2‑mal täglich für 7 Tage, danach Azithromycin 1 g 1‑malig, gefolgt von 500 mg 1‑mal täglich für 2 Tage
	*Indikation:* Bei nachgewiesener Makrolidresistenz oder Therapieversagen mit Azithromycin	
ÖGSTD*	k. A.	1) Azithromycin 500 mg an Tag 1, gefolgt von 250 mg 1‑mal täglich an Tag 2 bis 5 2) Josamycin 500 mg 3‑mal täglich für 10 Tage
*DSTIG*	Moxifloxacin 400 mg p.o. 1‑mal täglich für 7 bis 10 Tage	Doxycyclin 100 mg 2‑mal täglich für 7 Tage, danach Azithromycin 500 mg an Tag 1, gefolgt von 250 mg 1‑mal täglich an Tag 2 bis 5 (oder Azithromycin 1,5–2 g als ED) Pristinamycin 1 g 4‑mal täglich p.o. für 10 Tage (derzeit nur als Import aus Frankreich erhältlich)

* Derzeit in Überarbeitung

Die Behandlung asymptomatischer Infektionen wird nicht empfohlen und ist im Einzelfall Ermessenssache. Aufgrund der möglichen relevanten Morbidität bei Frauen und Männern, die Sex mit Frauen haben, nach Sicht der Autoren jedoch behandelt werden, bis neuere Daten zum natürlichen Verlauf der MG-Infektionen vorliegen [25].

In der Zurich Primary HIV Infection Study zeigte sich bei MSM eine auffallend hohe Prävalenz asymptomatischer MG-Infektionen (93 % der Fälle waren asymptomatisch), wobei die Mehrheit der Infektionen rektal, gefolgt von urethral detektiert wurde. Darüber hinaus wies ein hoher Anteil der Isolate eine genotypische Makrolidresistenz auf (73,3 %). In etwa 30 % der Fälle wurde eine spontane Clearance beobachtet. Angesichts der hohen Raten asymptomatischer und teils selbstlimitierender Infektionen wird eine zurückhaltende Therapieindikation empfohlen. Bei asymptomatischen Infektionen sollte eine Resistenztestung als Standard gelten; falls diese nicht verfügbar ist, stellt Moxifloxacin die bevorzugte Therapieoption dar [58].

## Fazit für die Praxis

- *Neisseria gonorrhoeae*: Es besteht eine hohe Rate an Resistenzen gegen Fluorchinolone, Penicillin und Tetracycline und zunehmend auch gegen Azithromycin. Ceftriaxon-Resistenzen bleiben im deutschsprachigen Raum selten und treten am ehesten bei Reiserückkehrer*innen (aus dem asiatisch-pazifischen Raum, insbesondere Südostasien und Japan) auf.
- *Mycoplasma genitalium*: Makrolidresistenzen finden sich bei etwa zwei Drittel aller MSM mit einer rektalen und/oder urethralen MG-Infektion.
- Diagnostik: NG und MG werden primär mittels NAATs nachgewiesen. Bei NG ist eine zusätzliche Kultur zur Resistenztestung anzustreben.
- Therapie NG: Als Erstlinientherapie wird eine Monotherapie mit Ceftriaxon 1–2 g i.m. oder i.v. empfohlen.
- Therapie einer symptomatischen MG-Urethritis: Empfohlen ist eine sequenzielle Therapie mit Doxycyclin, gefolgt von Azithromycin oder Moxifloxacin je nach Verfügbarkeit/Ergebnis einer Resistenzanalyse.
- Rationaler Antibiotikaeinsatz: Antibiotika sollten nur gezielt eingesetzt werden, um weitere Resistenzentwicklung zu vermeiden.
